# A lead-halide perovskite molecular ferroelectric semiconductor

**DOI:** 10.1038/ncomms8338

**Published:** 2015-05-29

**Authors:** Wei-Qiang Liao, Yi Zhang, Chun-Li Hu, Jiang-Gao Mao, Heng-Yun Ye, Peng-Fei Li, Songping D. Huang, Ren-Gen Xiong

**Affiliations:** 1 Ordered Matter Science Research Center, Southeast University, Nanjing 211189, China; 2 Fujian Institute of Research on the Structure of Matter, The Chinese Academy of Sciences, Fuzhou, Fujian 350002, China; 3 Department of Chemistry and Biochemistry, Kent State University, Kent, Ohio 44240, USA

## Abstract

Inorganic semiconductor ferroelectrics such as BiFeO_3_ have shown great potential in photovoltaic and other applications. Currently, semiconducting properties and the corresponding application in optoelectronic devices of hybrid organo-plumbate or stannate are a hot topic of academic research; more and more of such hybrids have been synthesized. Structurally, these hybrids are suitable for exploration of ferroelectricity. Therefore, the design of molecular ferroelectric semiconductors based on these hybrids provides a possibility to obtain new or high-performance semiconductor ferroelectrics. Here we investigated Pb-layered perovskites, and found the layer perovskite (benzylammonium)_2_PbCl_4_ is ferroelectric with semiconducting behaviours. It has a larger ferroelectric spontaneous polarization *P*_s_=13 μC cm^−2^ and a higher Curie temperature *T*_c_=438 K with a band gap of 3.65 eV. This finding throws light on the new properties of the hybrid organo-plumbate or stannate compounds and provides a new way to develop new semiconductor ferroelectrics.

## Abstract (web summary)

Lead-halide perovskite compounds have seen a considerable interest for their optoelectronic properties. Here, the authors discover a ferroelectric halide perovskite compound as an alternative pathway towards designing semiconductor ferroelectrics.

## Graphical abstract

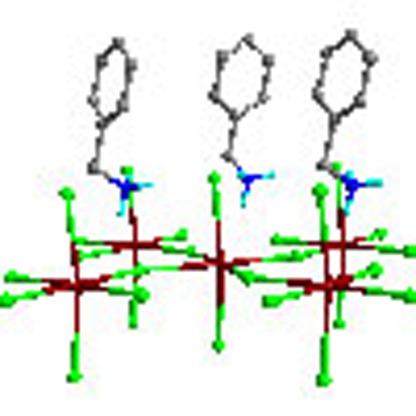

Hybrid organic–inorganic materials, as pointed out by Mitzi *et al*.^1^ in their pioneering work, have the potential of combining distinct properties of organic and inorganic components within a single molecular composite. In the wide field of hybrids, hybrid halometallates of Sn(II) or Pb(II) represent a technologically important class of semiconducting materials, which display remarkable electronic and optical properties. These hybrids adopt perovskite-like structures ranging from one dimension to three dimensions depending on the template organic ammonium cations^2^,^3^. The band gap of these semiconductors can be easily tuned over a wide range of energy by controlling the dimensionality of the inorganic framework or by modifying the halogen and/or the organic cations, which leads to their excellent electronic properties, including electrical conductivity^1^,^4^, ionic conductivity^5^, photoconductivity^6^, photo- and electroluminescence^7^,^8^,^9^ and exciton effect^9^,^10^,^11^. These properties make the hybrids great candidates for applications in optoelectronic devices, ranging from thin-film field-effect transistors^12^ to electroluminescent devices^13^ and to light absorbers of dye-sensitized solar cells^14^,^15^,^16^,^17^,^18^,^19^,^20^,^21^,^22^,^23^,^24^,^25^,^26^.

Structurally, hybrid halometallates of Sn(II) or Pb(II) are also suitable for exploring ferroelectricity, since they preserve the structural characteristics of perovskites. Especially, they are comprised of a variety of organic ammonium cations occupying the cavities enclosed by the MX_6_ octahedra giving rise to large freedom of motion. Such dynamic components are key elements in the design of molecule-based ferroelectrics, as exemplified by recently reported one- or three-dimensional organic–inorganic ferroelectrics such as (3-pyrrolinium)(CdCl_3_) (ref. 27) and (NH_4_)Zn(HCOO)_3_ (ref. 28). Inorganic semiconductor ferroelectrics such as BiFeO_3_ have shown great potential in applications in photovoltaics^29^ or photocatalytics^30^ owing to large photovoltage exceeding several times the band gap or switchable photocurrent. However, the extremely low power conversion efficiency, most of which is in the range of 10^−4^ or less^31^, limits its real application. Combination of ferroelectricity with the excellent carrier accumulation and transport in hybrid halometallates of Sn(II) or Pb(II) would lead to high-performance optoelectronic devices. Scientists have been aware of this possibility for a few years. For example, the excellent performance of CH_3_NH_3_PbI_3_ in solar cell application is thought to be related to its ferroelectric domain structures. However, up to now there has been no conclusive evidence for ferroelectricity in these compounds^32^,^33^,^34^. It is challenging to design molecular ferroelectrics with semiconducting properties.

We have recently designed an above-room-temperature perovskite one-dimensional organo–metal halide perovskite-like ferroelectric, which displays an anomalous photovoltaic effect with an open voltage of 32 V^27^. However, it shows a wide band gap of 4–5 eV, which is too high to achieve an optimum conversion efficiency of solar energy. As a continuation of our search for new molecular ferroelectric semiconductors with high Curie temperatures based on the Curie symmetry principle^35^, physical properties being parent groups of crystal structures, we searched the Cambridge Crystallographic Data Centre for compounds containing Pb ions, and found that catena-(bis(benzylammonium) tetrachloro-lead), (benzylammonium)_2_PbCl_4_ (**1**) crystallizes in a ferroelectric space group *Cmc*2_1_ (ref. 36). Systematic characterization reveals that compound **1** is a ferroelectric semiconductor. Herein we report the structural phase transition and corresponding dielectric, ferroelectric, polarization-switching and semiconducting properties of **1**.

## Results

### Structural phase transition

**Figure 1:**
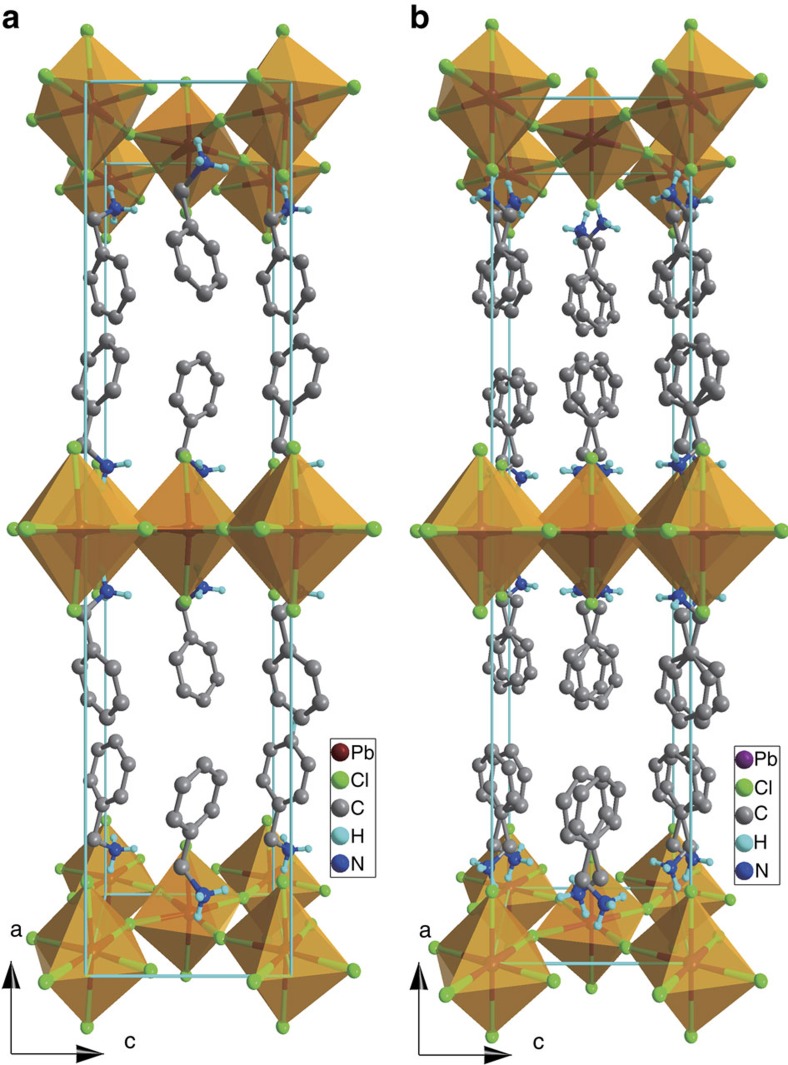
Comparison of the crystal structures between the paraelectric and ferroelectric phases. (**a**) Perspective view of **1** at 293 K. (**b**) Perspective view of **1** at 453 K. Hydrogen atoms bonded to the C atoms were omitted for clarity.

Thermal anomalies and changes of the second-order nonlinear susceptibility of the powder samples (see below), as well as large dielectric anomalies of the powder-pressed pellets (see Supplementary Fig. 1) at around *T*_c_=438 K suggest a reversible paraelectric-to-ferroelectric phase transition. To understand the structure–property relationship, we determined a series of structures at various temperatures (for crystal data, see Supplementary Table 1). The structures below *T*_c_=438 K are almost identical. We here take the structure determined at 293 K as the average structure of the low-temperature phase (LTP) and the structure at 453 K as the high-temperature phase (HTP). The structure at 293 K was refined in the polar space group *Cmc*2_1_, as reported in the literature^36^. The crystal structure can be described as the organic–inorganic-layered perovskite-like structure with the general formula A_2_BX_4_ (A is a monovalent organic ammonium, B a divalent transition metal and X a halogen), consisting of infinite, staggered layers of corner-sharing MCl_6_ octahedra interleaved by organic ammonium cations^37^,^38^. From the aspect of polarization, the characteristic feature of the structure is that all the C–N bonds of benzylammonium cations align along the *c* axis (Fig. 1a). One would expect that such alignment may exist in other halide analogues. We thus prepared the bromide analogue (benzylammonium)_2_PbBr_4_, and found it to be centrosymmetric in the temperature range 93–453 K (see Supplementary Table 2 and Supplementary Figs 2 and 3). The iodide analogue, (benzylammonium)_2_PbI_4_, is also centrosymmetric with space group *Pbca*^39^. Although the LTP is definitely non-centrosymmetric, as supported by the second-harmonic generation (SHG) and ferroelectric activity, the structures were alerted for a (pseudo) centre of symmetry by PLATON^40^, and the suggested space group is the *Cmca*. We tried the refinement of the structure at 423 K with the space group *Cmca*. The refinement converges; the obtained model is similar to that in the HTP (Fig. 1b). These indicate that the LTP especially those near *T*_c_ probably contain partially disordered organic cations.

The cell constants of the HTP at 453 K approximate to those in the LTP, respectively. The structure was refined in the space group *Cmca*, as suggested by SHELXTL^41^, which is consistent with the pseudo symmetry of the LTP as well as the non-activity in the SHG response. The Pb layers show no significant modification, while the benzylammonium cation shows an obvious change in the arrangement. It is located on the crystallographic twofold rotation axis normal to the *bc*-plane, and thus was modelled a as twofold orientational disorder (Fig. 1b). Accordingly, the molecular electric dipole moment along the *c* axis cancels each other out. As shown in Fig. 1, one of the two orientations in the HTP is the same as that in the LTP. Thus, the formation of the ferroelectric LTP and the ferroelectric polarization reversal (see below) can be understood in terms of the reorientation of the benzylammonium cations by rotation between two potential minima.

### Dielectric properties

**Figure 2:**
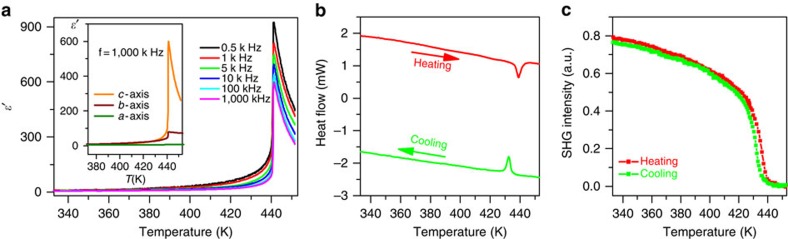
Dielectric, thermal and SHG properties of 1. (**a**) Temperature dependence of the real part (*ɛ*′) of the complex permittivity measured along the *c* axis at different frequencies. Inset: comparison of real parts measured along different directions. (**b**) DSC curves in the heating and cooling runs. (**c**) Temperature dependence of the second-order nonlinear optical coefficient of **1** measured on crystalline samples.

There is no exception to the rule that the physical properties for a transition from one phase to another phase will display an anomaly near *T*_c_ as a response to external stimuli such as pressure, temperature, electric and magnetic fields. It is an easy way to detect whether there exists a sharp peak at *T*_c_ in the measurements of the temperature dependence of permittivity. As shown in Fig. 2a, the permittivity *ɛ*′ (the real part (*ɛ*′) of the complex dielectric constant *ɛ*=*ɛ*′−*iɛ*″, where *ɛ*″ is the imaginary part) as a function of temperature shows sharp peaks at *T*_c_=438 K at different frequencies. *T*_c_ is close to or even higher than those for our recently reported high-temperature molecular ferroelectrics^42^,^43^. The peak values ranging from 600 to 900 are a few hundred times larger than those in the stable states respectively, which is the main characteristic of a ferroelectric transition. The permittivity at a lower frequency is somewhat larger than that at a higher frequency. The permittivity shows significant anisotropy. As illustrated in the inset of Fig. 2a, the dielectric measured along the *a* axis shows no obvious anomaly at around *T*_c_, and that along the *b* axis has an enhancement of a few times, while that along the *c* axis exhibits an enhancement of a few hundred times due to the appearance of spontaneous polarization in this direction.

### Differential scanning calorimetry

Similarly, the structural phase transition is accompanied by a thermal anomaly at around *T*_c_. As shown in Fig. 2b, differential scanning calorimetry (DSC) measurements definitely demonstrate a peak at around *T*_c_, in good agreement with the measurements of the temperature-dependent dielectric constant. The DSC curve shows peaks at 438 and 433 K in the heating and cooling runs, respectively. The total entropy gain ΔS=1.098 Jmol^−1^K^−1^ is close to Rln1.14 (R is the gas constant), inconsistent with the model of order–disorder transition in the structural determination. This means that the ordering of the organic cations is completed in a wide temperature range, as discussed in the section of structural phase transition.

### Second-harmonic generation

As only non-centrosymmetric materials are SHG-active, it is a sensitive tool for probing the loss of inversion symmetry during a phase transition. In this case, as temperature increases, *χ*^(2)^ shows a gradual decrease at around *T*_c_ (Fig. 2c). It is very important to observe that above *T*_c_, *χ*^(2)^ is almost zero, probably suggesting that the paraelectric phase is centrosymmetric, which supports the crystal structure determination at 453 K. The change of SHG signal is reversible, showing almost overlapping curves in the heating and cooling runs. Thus, it is evident that symmetry breaking occurs during the paraelectric to ferroelectric phase transition, losing inversion and mirror symmetries. It is convenient to calculate how many symmetry elements are lost, which are, in detail, *i*, and *C*_2_′, *C*_2_′ and σ_*h*_ during the transition from the paraelectric phase (point group *D*_2*h*_) to the ferroelectric phase (point *C*_2*v*_).

### Ferroelectric and pyroelectric properties

**Figure 3:**
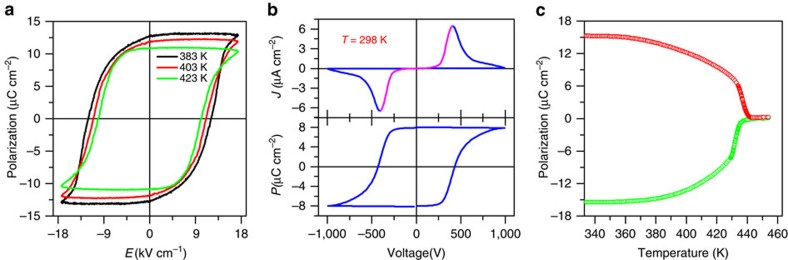
Properties of polarization switching for **1**. (**a**) *P*∼*E* hysteresis loops measured at different temperatures along the *c* axis by the Sawyer-Tower circuit method. (**b**) *P*∼*E* hysteresis loops measured along the *c* axis at room temperature by using the double-wave method. (**c**) Polarization as a function determined by the measurement of the pyroelectric effect.

The direct proof of ferroelectricity is the observation of a typical polarization electric field (*P*∼*E*) hysteresis loop. Figure 3a illustrates *P*∼*E* hysteresis loops measured by the Sawyer-Tower circuit^44^ method. It reveals that the polarization switching can be reached at higher temperatures. The switching cannot be observed at room temperature, because of the quick increase of coercive electric field (*E*_c_) with a decrease in temperature. Thus, we only measured *P*∼*E* loops at above 383 K using this method. The estimated saturated polarization (*P*_s_) is about 13 μC cm^−2^ with a relatively higher *E*_c_. *P*_s_ is among the highest values observed for molecular ferroelectrics^42^,^43^,^45^,^46^,^47^,^48^,^49^,^50^. This value is also close to that determined by the measurement of pyroelectricity (Fig. 3c). The remnant polarization (*P*_r_) is almost equal to *P*_s_. Interestingly, if measured by the double-wave method^51^,^52^, the *P*∼*E* hysteresis loop can be obtained at room temperature. There are two peaks in the *J*∼*V* curve, as shown in Fig. 3b. The two opposite peaks indicate two stable states with opposite polarity. According to the current accumulating, a *P*∼*E* hysteresis loop has been obtained at room temperature. The measured *P*_s_ with the double-wave method is much smaller than that with the Sawyer-Tower circuit method, probably suggesting that not all the dipolar moments can be reversed under an external electric field. The part of the *J*∼*V* curve between the two peaks of *J* resembles the *I*∼*V* curve for a semiconductor, which is why ferroelectric materials can be used as field effect transistors.

### Piezoresponse force microscopy

**Figure 4:**
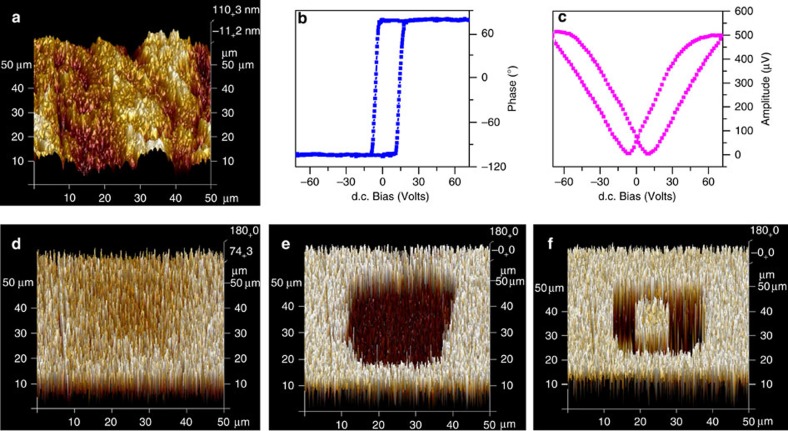
Polarization-switching properties of **1** investigated by piezoresponse force microscopy (PFM) imaging. (**a**) Topographic image of the crystal surface of **1**. (**b**,**c**) Local PFM hysteresis loops measured in the crystal surface ((**b**) phase signal and (**c**) amplitude signal). (**d**) PFM image of the as-grown crystal surface; the white region and pale-yellow region contrast probably indicates the regions with polarization oriented downwards. (**e**,**f**) PFM images of a polarization pattern produced by scanning with the tip under ±72 V, respectively (dark-brown region corresponds to downward polarization; white with little yellow region represents upward polarization).

To further understand the polarization-switching behaviours, we performed the switching measurements using an atomic force microscope (Brucker Multimode 8) with a resonant-enhanced piezoresponse force microscopy mode to obtain images of polarization patterns in a bulk crystal surface of **1,** similar to others^53^,^54^,^55^,^56^. Figure 4a demonstrates the topographic image of the crystal surface of **1**; Fig. 4b,c shows a local piezoresponse force microscopy response of the crystal surface of **1**, revealing a hysteretic behaviour typical for ferroelectric polarization switching. Note that the hysteresis loop is characterized by a shift towards a positive voltage up to 72 V. The direction of the field is upward (that is, from the bottom of the bulk crystal to the tip), which is parallel to the direction of the polarization of the bulk crystal surface of **1**. To demonstrate polarization-switching behaviours, we switched the upward-polarized single-domain state of the bulk crystal surface of **1** (Fig. 4d) to a bidomain-patterned state (Fig. 4e). For this purpose, polarization in a 30 × 30-μm^2^ square region of the crystal surface was switched downwards by scanning the crystal surface of **1** with a tip biased with a voltage *V*_tip_=−72 V that exceeds the coercive voltage for the crystal surface. Then, polarization within an area of 10 × 10 μm^2^ in the centre of the square area was switched back (upwards) by applying a bias *V*_tip_=72 V to the tip (Fig. 4f). Figure 4e,f clearly demonstrates the resulting polarization pattern in which antiparallel domains, and reversible polarization behaviours such as upward- and downward-direction writings are demonstrated in different colours. This reveals that the domains in the crystal surface of **1** can be switched by an external field.

### Semiconducting properties

**Figure 5:**
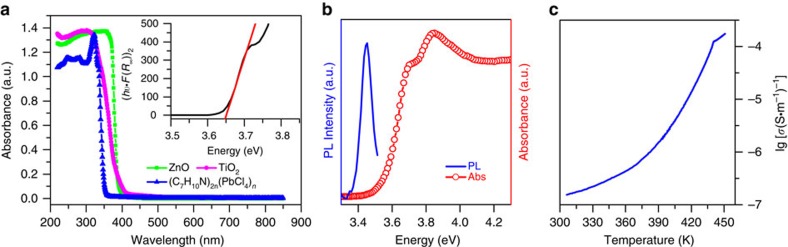
Semiconducting properties of **1**. (**a**) Ultraviolet−vis absorption spectra of **1**, TiO_2_ and ZnO. The inset shows the Tauc plot for **1**. (**b**) Room-temperature photoluminescence spectrum. (**c**) Temperature dependence of the a.c. conductivity of **1** at the frequency 500 Hz.

To understand the semiconducting properties of **1**, we performed optical ultraviolet–visible (vis) absorption spectrum measurements. The optical absorption characteristics of semiconductors are highly related to its band gap type, direct band gap or indirect band gap. The direct band gap semiconductor ZnO and the indirect band gap semiconductor anatase-TiO_2_ were used for comparison. As shown in Fig. 5a, unlike anatase-TiO_2_ but similar to ZnO, **1** clearly displays a sharp absorption edge in the ultraviolet spectral region, suggesting a direct band gap behaviour. The optical band gap can be determined by the variant of the Tauc equation:^57^


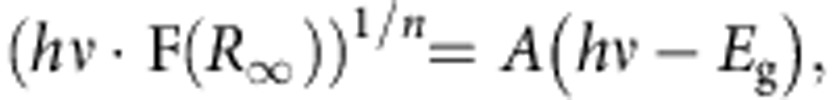


Where *h* is the Planck's constant, *ν* is the frequency of vibration, F(*R*_∞_) is the Kubelka−Munk function^58^, *E*_g_ is the band gap and *A* is the proportional constant. The value of the exponent *n* depends on the nature of the sample transition, *n*=1/2 for a direct allowed transition, while *n*=2 for indirect allowed transition. Hence, the band gap *E*_g_ can be obtained from a Tauc plot (inset of Fig. 5a) of (*hν*·F(*R*_∞_))^1/*n*^ versus the energy in eV by extrapolating the linear region to the *x* axis intercept. The estimated band gap *E*_g_ is 3.65 eV for **1**, comparable to other analogues with the general formula (R–NH_3_)_2_PbCl_4_ (ref. 59), 3.20 eV for anatase-TiO_2_ and 3.29 eV for ZnO. At the same time, a strong ultraviolet emission was also observed at the vicinity of the onset of the absorption edge (Fig. 5b), with a Stoke shift of ∼200 meV.

The temperature dependence of the a.c. conductivity of the crystal of **1** along the *c* axis measured at frequency 500 Hz is shown in Fig. 5c. The positive slope of the a.c. conductivity with increasing temperature in the room-temperature phase is the characteristic of a semiconductor. The discontinuity of the conductivity at around *T*_c_, which increased by a factor of about 10^2^, corresponds to the structural phase transitions. These behaviours, that is, the steep increase of absorbance at the band edge in the ultraviolet–vis spectra and the conductivity increases exponentially with the temperature increasing, reveal that **1** is a typical semiconductor.

## Discussion

**Figure 6:**
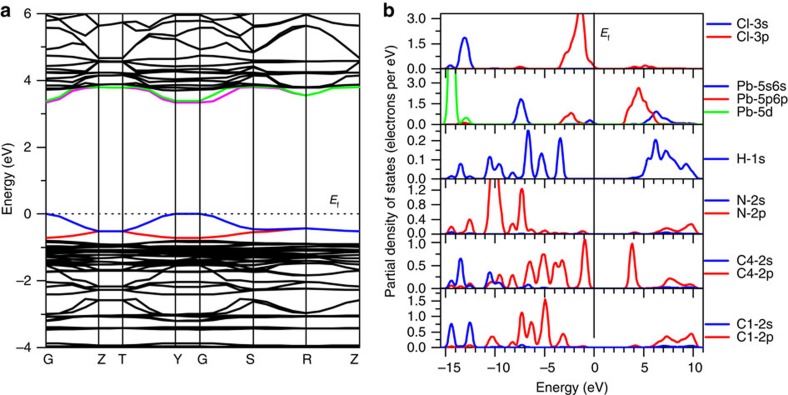
The calculated band structure. (**a**) The partial density of states (PDOS). (**b**) The PDOS of all C atoms in benzene ring is very similar, so we take the C4 atom as an example to exhibit the PDOS of the C atoms in the benzene ring.

We calculated the band structure of **1** based on density functional theory (DFT) to understand the electronic mechanism of the semiconducting properties. The calculated band structure is displayed in Fig. 6a. Both the conduction band (CB) minimum and the valence band (VB) maximum are localized at the G point, so it is a direct band-gap semiconductor; and the calculated band gap is 3.34 eV, slightly smaller than the experimental value of 3.65 eV, due to the limitation of the DFT methods^60^,^61^,^62^.

The bands can be assigned according to the partial density of states (PDOS), as plotted in Fig. 6b. From PDOS, it is obvious that for the organic part, H-1s states overlap fully with C-2p and N-2p states over almost the whole energy region, indicating the strong covalent interactions in C–H and N–H bonds. In the vicinity of the Fermi level (*E*_f_) of PDOS, there are two peaks localized around −0.95 and 3.82 eV, which correspond to the flat bands in the same energy regions of the band structure. They mainly originate from the π and π* of C–C bonds in benzene rings. For the inorganic part, in almost the whole energy region (−12∼−15 eV, −6.5∼−8 eV, −3.5∼0 eV and 3.0∼6.3 eV), Pb-s/p/d and Cl-s/p states overlap obviously, showing the strong interactions between Pb and Cl atoms. The dispersed bands at the VB top and the CB bottom in the band structure are plotted in colour and the corresponding orbitals graphs are displayed in Supplementary Fig. 4. The bands at the VB top are originated from the nonbonding states of Cl-3p, and those at the CB bottom are mainly from the unoccupied Pb-6p orbitals. Clearly, both VB maximum and CB minimum are from the electronic states of Pb and Cl atoms, so it is the inorganic part to determine the band gap of the material.

In summary, we have successfully demonstrated that the hybrid organo-plumbate (benzylammonium)_2_PbCl_4_ (**1**) is a high-temperature molecular ferroelectric with semiconductive behaviours. Although **1** has a relatively high band gap, the tunability of hybrid organo-plumbate surely guarantees that the band gap and visible absorbance characteristics can be tailored towards the potential applications in photovoltaics through cation and halide replacement. Work on enhancing such properties is currently underway.

## Methods

### Crystal growth

**1** was prepared as small crystals by mixing a stoichiometric amount of PbCl_2_ and benzylammonium chloride in a concentrated HCl aqueous solution. Large crystals up to 5 × 10 × 2 mm^3^ were obtained by slow evaporation of a N,N-dimethylformamide (DMF) solution at 363 K. The large crystals are plate-like, and have a morphology as modelled from Material Studio. They are elongated along [0 0 1] direction, while the largest face is the (1 0 0) face (see Supplementary Fig. 5). The purity of the bulk phase was verified by powder X-ray diffraction and infrared spectra (see Supplementary Figs 6 and 7). The thermal stability of **1** is maintained up to at about 480 K (see Supplementary Fig. 8).

### Physical properties measurement

Methods of DSC, SHG, dielectric, pyroelectric, *P*∼*E* hysteresis loop measurements were described elsewhere^47^,^63^. For dielectric, pyroelectric, *P*∼*E* hysteresis loop measurements, single-crystal plates with 5 mm^2^ in area and 0.5 mm in thickness were cut from the large crystals in the [0 0 1] direction. Silver conduction paste deposited on the plate surfaces was used as the electrodes.

### Ultraviolet−vis spectra

Ultraviolet–vis diffuse-reflectance spectra measurements were performed at room temperature using a Shimadzu UV-2450 spectrophotometer mounted with ISR-2200 integrating sphere operating from 220 to 850 nm. BaSO_4_ was used as a 100% reflectance reference. Powdered crystals of **1** were prepared for measurement. The generated reflectance-versus-wavelength data were used to estimate the band gap of the material by converting reflectance data to absorbance according to the Kubelka−Munk equation: F(*R*_∞_)=(1−*R*_∞_)^2^/2*R*_∞_. The optical band gaps of **1**, TiO_2_ and ZnO were determined by plotting (*hν*·F(*R*_∞_))^1/*n*^ against the energy in eV and extrapolation of the linear region to the *x*-axis intercept.

### A.c. conductivity

The a.c. conductivity *σ*_a.c._ of single-crystal **1** along the *c* axis at frequency 500 Hz was obtained from the imaginary part of the dielectric permittivity *ɛ"* by using the relation: *σ*_a.c._=*ωɛ"ɛ*_0_, where *ω* is the angular frequency 2*πf* and *ɛ*_0_ is the permittivity of free space (8.854 × 10^−12^ F m^−1^).

### Computational descriptions

Single-crystal structural data at 293 K of compound **1** was used for the theoretical calculations. The electronic structures calculations, including band structure, DOS and frontier orbitals, were performed by the DFT method within the total-energy code CASTEP^64^,^65^. The exchange and correlation effects were treated by Perdew–Burke–Ernzerhof in the generalized gradient approximation^66^. The core-electrons interactions between the ionic cores and the electrons were described by the norm-conserving pseudopotential with the following valence electron configurations: Pb-5s^2^5p^6^5d^10^6s^2^6p^2^, Cl-3s^2^3p^5^, C-2s^2^2p^2^, N-2s^2^2p^3^ and H-1s^1^ (ref. 67). In addition, a Monkhorst−Pack *k*-point sampling of 3 × 3 × 3 and an energy cutoff of 820 eV were adopted in our calculation.

## Additional information

**Accession codes**: The structures have been deposited at the Cambridge Crystallographic Data Centre (deposition numbers: CCDC 1042742-1042752).

**How to cite this article:** Liao, W.-Q. *et al*. A lead-halide perovskite molecular ferroelectric semiconductor. *Nat. Commun*. 6:7338 doi: 10.1038/ncomms8338 (2015).

## Supplementary Material

Supplementary Information Supplementary Figures 1-8 and Supplementary Tables 1-2
