# Supplementary material for: A lead-halide perovskite molecular ferroelectric semiconductor
Source: Nat Commun. 2015 May 29;6:7338. doi: 10.1038/ncomms8338 (PMC4458893; doi:10.1038/ncomms8338)
Supplement: Supplementary Information — Supplementary Figures 1-8 and Supplementary Tables 1-2 [file ncomms8338-s1.pdf]

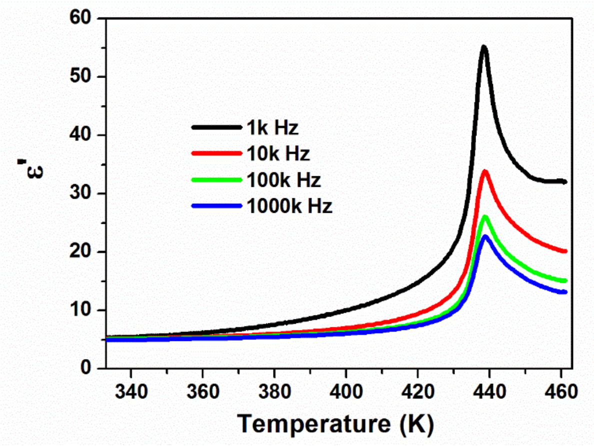

Supplementary Figure 1. Permittivity of (benzylammonium)$_2$PbCl$_4$ (**1**) as function of temperature measured on a powder-pressed pellet, showing relatively larger dielectric anomalies.

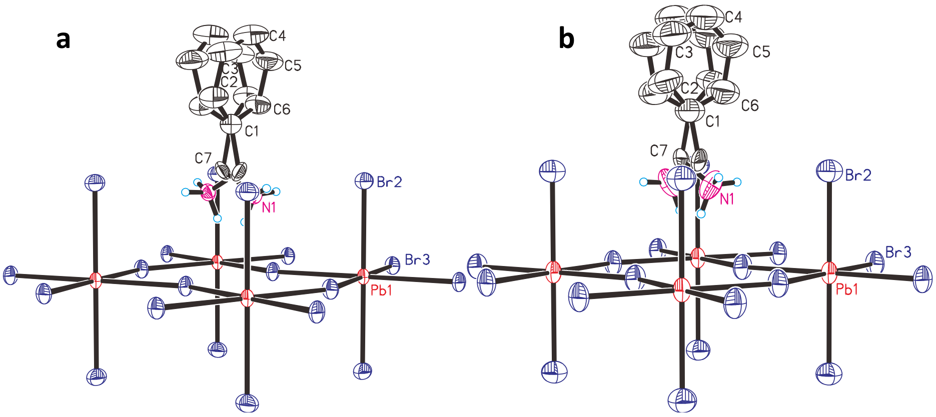

Supplementary Figure 2. Asymmetric units of (benzylammonium)$_2$PbBr$_4$ (**2**) at various temperatures, showing the similarities. (**a**) Ellipsoid drawing of asymmetric unit of **2** at 298 K. (**b**) Ellipsoid drawing of asymmetric unit of **2** at 423 K. H-atoms bonded to C-atoms were omitted for clarity. The non-hydrogen atoms not labeled were generated by symmetry operation. Displacement ellipsoids were drawn at the 30% probability level

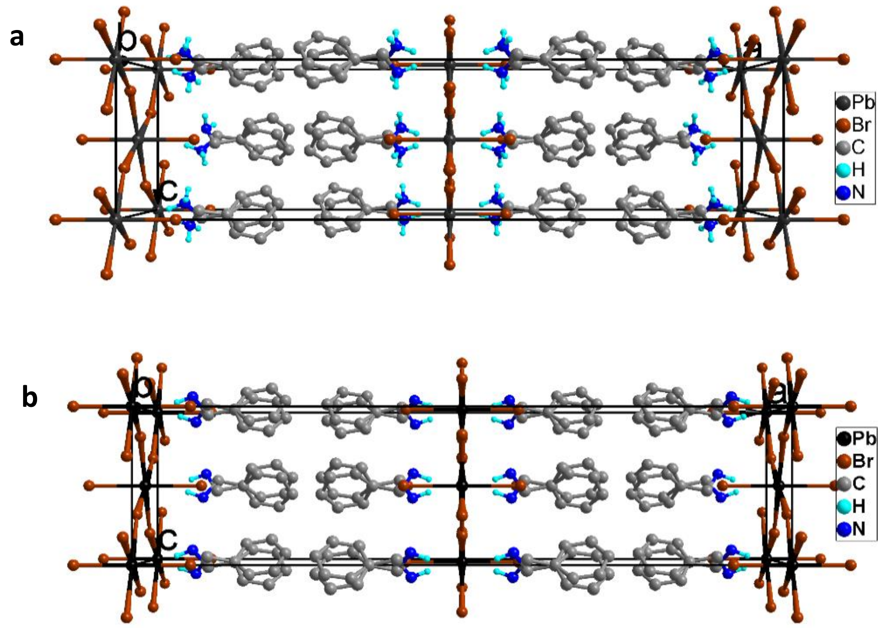

Supplementary Figure 3. Crystal structures of $(benzylammonium)_2PbBr_4$ at various temperatures, showing the similarities. (**a**) A packing view of $(benzylammonium)_2PbBr_4$ (**2**) at 293 K. (**b**) A packing view of **2** at 423 K. Hydrogen atoms bonded to the C atoms were omitted for clarity. For all structures, the systemic absence at 298, 323, 373 and 423 K are almost the same. The suggested spacegroups are *Cmca* and *Aba*2, and the more possible spacegroup is the *Cmca* because the mean value of $|E^2-1|$ for each temperature structure is close to the theoretically expected value of 0.968 for a centrocsymmetric structure. Since the cell constants of $(benzylammonium)_2PbBr_4$ is similar to those of $(benzylammonium)_2PbCl_4$, one would expect the same space group for $(benzylammonium)_2PbBr_4$ and $(benzylammonium)_2PbCl_4$. At this stage, we sorted to the SHG response. We performed SHG measurements in the temperature range 298-453 K. No double frequency signal was observed. Thus, we finally refined all these structure in the centrosymmetric spacegroup *Cmca.* As shown in supplementary Figs 2 and 3, the final models are almost the same.

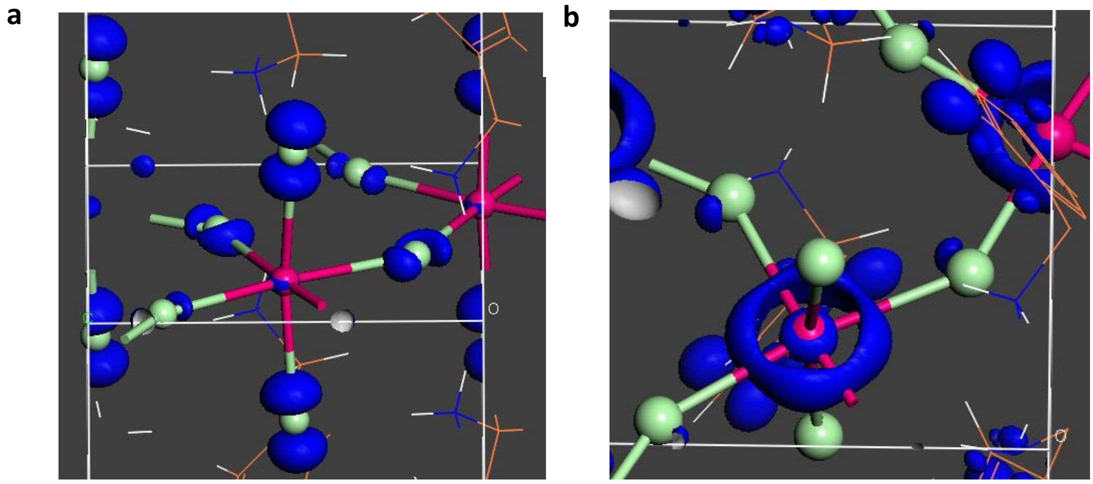

Supplementary Figure 4. Calculated molecular orbitals. (**a**) Calculated molecular orbital located at the valence band. (**b**) Calculated molecular orbital at the conduction band.

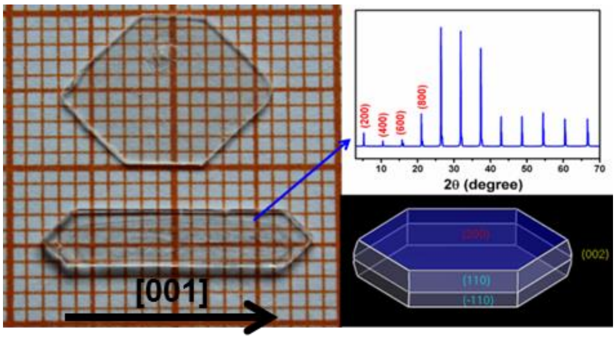

Supplementary Figure 5. An image of two large single crystals of (benzylammonium)$_2$PbCl$_4$ (**1**), and the simulated morphology from Material Studio.

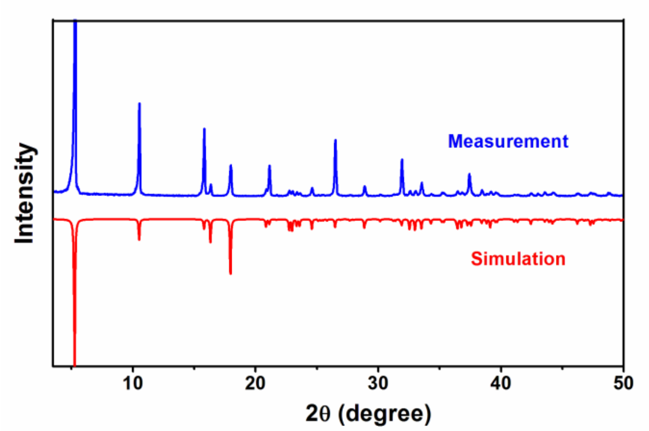

Supplementary Figure 6. Pattern of powder diffraction for (benzylammonium)$_2$PbCl$_4$ (**1**), verifying the purity of the bulk phase.

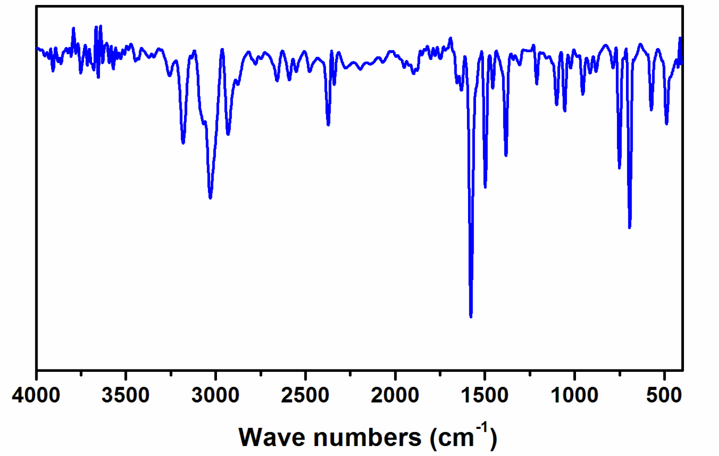

Supplementary Figure 7. The Infrared spectrum for (benzylammonium)$_2$PbCl$_4$ (**1**).

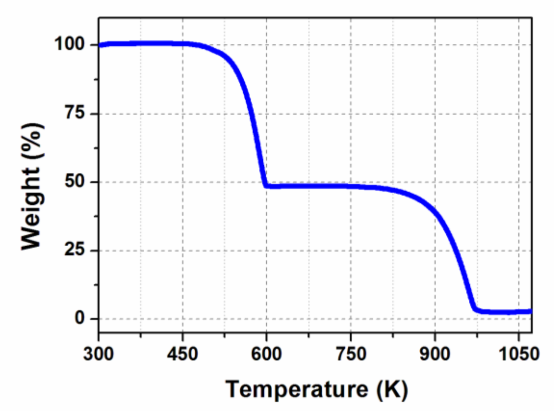

Supplementary Figure 8. Thermal analysis for (benzylammonium)$_2$PbCl$_4$ (**1**), showing the stability up to about 480 K.

Supplementary Table 1. Crystal data for (benzylammonium)$_2$PbCl$_4$ (for all structures: Mr = 565.31, Z = 4).

	93 K	173 K	293 K	323 K	373 K	423 K	453 K
Chemical formula				$(C_7H_{10}N)_2PbCl_4$			
Crystal system, space group	orthorhombic, *Cmc*2$_1$	orthorhombic, *Cmc*2$_1$	orthorhombic, *Cmc*2$_1$	orthorhombic, *Cmc*2$_1$	orthorhombic, *Cmc*2$_1$	orthorhombic, *Cmc*2$_1$	orthorhombic, *Cmca*,
a, b, c (Å)	33.150(16) 7.773(3) 7.652(4)	33.317(16) 7.785(3) 7.680(4)	33.64(2) 7.817(4) 7.737(4)	33.65(3) 7.806(8) 7.732(7)	33.91(4) 7.842(8) 7.776(9)	34.39(6) 7.920(14) 7.880(15)	34.62(13) 7.84(3) 7.94(3)
V (Å^3)	1971.5(16)	1991.9(16)	2034.3(19)	2031(3)	2067(4)	2146 (7)	2157(14)
Dc (g cm^{-3})	1.905	1.885	1.846	1.849	1.816	1.749	1.741
μ (mm^{-1})	9.095	9.001	8.814	8.827	8.8673	8.8353	8.8314
R_1 (I > 2σ(I))	0.0394	0.0375	0.0374	0.0453	0.0535	0.0588	0.0771
wR_2 (all data)	0.0674	0.0688	0.0666	0.0954	0.1272	0.1546	0.1545
S	0.929	0.921	0.889	0.955	0.944	0.956	1.201

Supplementary Table 2. Crystal data for (benzylammonium)$_2$PbBr$_4$ (for all structures: Mr = 743.1, Z = 4)

	293 K	323 K	373 K	423 K
Chemical formula			$(C_7H_{10}N)_2PbBr_4$	
Crystal system,	orthorhombic,	orthorhombic,	orthorhombic,	orthorhombic,
space group	*Cmca*	*Cmca*	*Cmca*	*Cmca*
a, b, c (Å)	33.394(17)	33.49(3)	33.78(5)	33.87(3)
	8.153(4)	8.169(6)	8.200(11)	8.183(7)
	8.131(4)	8.151(6)	8.198(12)	8.193(8)
V (Å^3)	2214(2)	2031(3)	2271(6)	2270(4)
*D*c (g cm^{-3})	2.230	2.213	2.174	2.174
μ (mm^{-1})	14.836	14.728	14.728	14.463
R_1 (I > 2σ(I))	0.0465	0.0445	0.0488	0.0487
wR_2 (all data)	0.1017	0.1027	0.1136	0.1176
S	1.009	0.982	1.083	0.891
